# How We Use Reticulocyte Parameters in Workup and Management of Pediatric Hematologic Diseases

**DOI:** 10.3389/fped.2020.588617

**Published:** 2020-12-04

**Authors:** Emilia Parodi, Francesca Romano, Ugo Ramenghi

**Affiliations:** ^1^Pediatric Hematology, Department of Pediatric and Public Health Sciences, University of Torino, Turin, Italy; ^2^Postgraduate School of Pediatrics, University of Torino, Turin, Italy

**Keywords:** reticulocyte, CHr, ARC, iron deficiency anemia, anemia, pediatric, newborn

## Introduction

Reticulocyte parameters are simple to obtain, require a very small extra amount of blood and provide additional information about erythrocyte production. However, their use in pediatric routine care is still limited.

Aim of the present opinion paper is to describe the usefulness of these tools for Pediatricians in the workup and management of pediatric hematologic diseases.

## Reticulocyte Parameters

### Reticulocyte Physiology

Reticulocytes are the youngest erythrocytes that the bone marrow releases into peripheral blood. Thus, they represent a reliable index of recent bone marrow erythrocyte production.

Under normal conditions, nucleated erythroid precursor cells clonally mature in 1–3 days within the bone marrow. When their nucleus is extruded from the cell, the young erythrocytes (i.e., reticulocytes) are released into peripheral blood where they circulate for 1–2 days, before becoming mature erythrocytes. Reticulocytes can be identified through specific staining (manual reticulocyte count) or through quantification of their residual RNA (automated reticulocyte count) (1).

### Absolute Reticulocyte Count (ARC)

Usually, the reticulocyte reference range is reported as a percentage of the red blood cells total. After the first few months of life, the normal reticulocyte percentage in children is equal to the adult's one (~1.5 percent) (2).

However, in patients with anemia, the reticulocyte percentage must be interpreted in relation to the reduced number of red blood cells. For example, in children affected by aplastic anemia, a normal reticulocyte percentage does not reflect a proper bone marrow production.

The absolute reticulocyte count (ARC), defined as the number of reticulocytes/μL, better reflects bone marrow function and effective erythropoiesis. A normal ARC both in pediatric (after the first 3 months of life) and adult age is ~25,000 to 75,000/μL (1.0 ± 0.5 percent of the 5 million red cells/μL) and is now calculated and reported by many automated cell counters (2).

### New Reticulocyte Parameters: The Reticulocyte Hemoglobin Content (CHr)

Modern analyzers and flow cytometric techniques, although not readily available in all institutions, can measure red blood cell and reticulocyte features more accurately than in the past, without relevant additional costs.

The reticulocyte hemoglobin content (CHr) is the product of the cellular volume and the cellular hemoglobin concentration. CHr is obtained on a very small sample of blood as part of the complete blood count and does not require additional phlebotomy (3). Since erythrocytes have a slow turnover (120 days), their indices are not efficient indicators of early iron deficient erythropoiesis. On the contrary, as reticulocytes circulate for only 1–2 days, CHr provides a reliable measure of the functional iron available for new red blood cell production over the previous 3–4 days and proved to be sensitive indicator of early iron deficient erythropoiesis.

### Reticulocyte Parameters in the Newborn

After birth and until 8–12 weeks, hemoglobin physiologically falls due to the suppression of erythropoietin in the extrauterine hyperoxic environment. This drop is more dramatic in preterm infants. At birth, the reticulocyte count is higher than at any time during healthy life; ARC abruptly falls during the first days after birth, following a fall in erythropoietin production. A slight upturning in reticulocyte count is expected in the subsequent 3 weeks; after the first 3 months of life ARC reaches the normal reference range for pediatric and adult population (4).

Even though there are no standardized cut offs in the newborn, CHr is a good marker of iron status also in the first days and months of life, even in preterm and critically ill babies.

## Discussion

We will review and discuss the usefulness of reticulocyte parameters in the diagnosis and management of pediatric hematologic diseases.

### Absolute Reticulocyte Count (ARC) in the Diagnosis of Pediatric Anemia

As the reticulocyte count distinguishes disorders resulting from rapid destruction or loss of erythrocytes from disorders resulting from bone marrow failure, we strongly suggest its use in the initial diagnostic workout of pediatric anemia.

✓ High reticulocyte count: consider hemolysis (i.e., red blood cells membrane defects, enzymatic defects, autoimmune diseases, sickle cell disease), or bleeding
✓ Low reticulocyte count: consider bone marrow depression (i.e., infections, transient erythroblastopenia of childhood, congenital bone marrow failures)
✓ Note: Parvovirus B19 can cause transient aplastic crisis in patients with chronic hemolytic diseases (i.e., spherocytosis, sickle cell disease, thalassemia trait, congenital dyserythropoietic anemia) resulting in severe acute anemia. In these patients, ARC is a very useful index in identifying transient marrow suppression and the need of transfusion.

### Absolute Reticulocyte Count (ARC) in the Follow up of Pediatric Anemia

ARC reflects both spontaneous bone marrow recovery (for e.g., after transient bone marrow suppression caused by a viral infection) and response to therapy in iron deficiency anemia.

We previously identified ARC as an accurate marker to early detect response to exclusive oral iron supplementation (iron sulfate 2 mg/kg/die) in a cohort of children with severe iron deficiency anemia. A significant association was demonstrated between relative increase of ARC at day +3 and Hb at day +14 (5).

**Figure 1:**
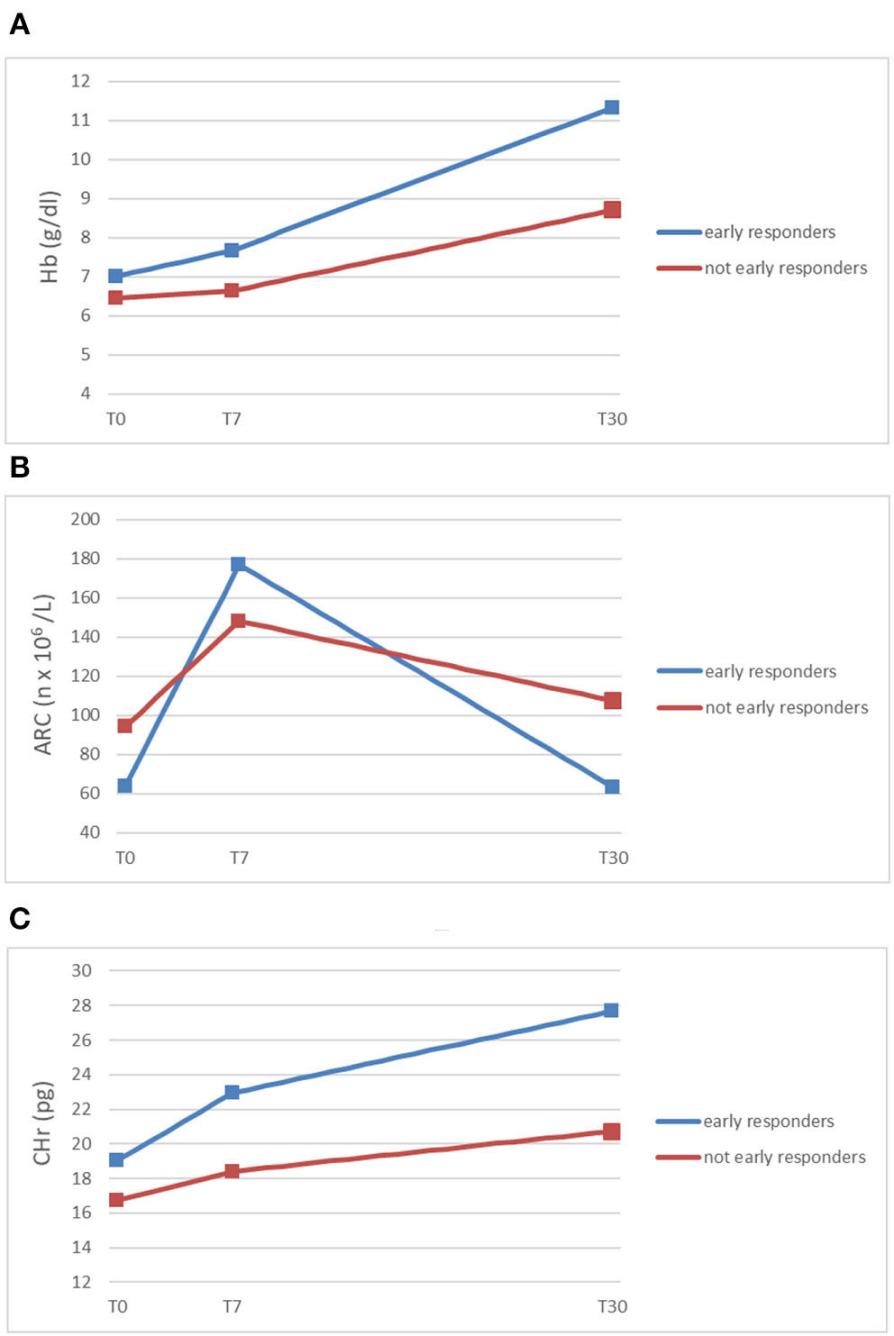
Trends of mean Hb **(A)**, ARC **(B)**, and CHr **(C)** (*y*-axis) in early responding patients and not early responding patients at diagnosis (T0), within 7 days from the beginning of oral iron supplementation (T7), and after 30 days (T30) (*x*-axis) (5, 6).

Subsequently, we demonstrated the relative increase of ARC at day + 7 to be predictive of complete early response (normal hemoglobin value per age and sex at day +30) (Figure 1) (6).

### Reticulocyte Hemoglobin Content (CHr) in the Diagnosis of Iron Deficiency Anemia

CHr has been validated in literature as the strongest independent predictor of iron deficiency and iron deficiency anemia, when compared to other parameters (hemoglobin, ferritin, transferrin saturation, or mean corpuscular volume–MCV), both in adults and children. In pediatric age, optimal CHr cut-off was established at 27.5 pg for detecting iron deficiency (sensitivity 83% and specificity 72%) (7) and 26 pg for detecting iron deficiency anemia (sensitivity 83% and specificity 75%) (8).

However, since MCV is used to calculate CHr, this index has diagnostic limitations in patients with other causes of altered mean MCV (it is low in iron-replete patients with hemoglobinopathies associated with microcytosis and it can be normal or elevated in iron-deficient patients with high MCV, for example in patients with both iron and Vitamin B12 deficiency) (9).

### Reticulocyte Hemoglobin Content (CHr) in the Follow Up of Iron Deficiency Anemia

CHr has been shown to be an accurate marker in monitoring response to iron therapy in adults and in pediatric patients receiving hemodialysis (3).

For the first time in literature, we demonstrated CHr to be the earliest marker of response to oral iron treatment (48 h) in children with severe iron deficiency anemia (5).

Moreover, CHr measured within 7 days from the start of oral iron resulted significantly higher in children who reached reference value of hemoglobin (Hb) concentration for age and sex at day +30 from the beginning of treatment (6).

As shown in Figure 1, ARC and CHr are early and accurate predictors of response to oral therapy in iron deficiency anemia. Early detection of patients not responding to oral iron (malabsorption? persistent blood loss?) is crucial to switch them to different diagnostic workout and/or treatment, such as parenteral iron supplementation or transfusion.

### Reticulocyte Parameters and the Newborn

Both anemia of prematurity and iron deficiency anemia in early stages of life can affect long term infants' growth and neurologic development. Thus, it is essential for the pediatrician to promptly detect and treat these conditions and to take advantage of easy tools like reticulocyte parameters also in the first days and months of life.

Iron stores in the newborn mainly depend on maternal stores, gestational age (most of the transfer of maternal iron to the fetus occurs during the third trimester of gestation) timing of cordon clamping [delayed cordon clamping, i.e., not earlier than 1 min after birth, improves iron status in the child (10)] and timing of introduction of complementary food. In term healthy children, iron is usually sufficient up to when the child has doubled its weight (2), which usually occurs between the 4th and the 6th month of life. The introduction of complementary food after 6 months of age exposes the child to iron deficiency especially in exclusive breastfed infants (11, 12). Particularly, late preterm babies represent an underestimated category at risk of iron deficient anemia (13).

CHr could be a simple and reliable index to detect iron deficiency both in the newborn and in late preterm infants and low birth weight infants on exclusive breastfeeding, especially in those with an important catch-up growth and delay in introduction of complementary food. Compared to other indices, it requires less blood and it is not affected by gestational age, stress, and inflammation.

Some authors suggested 25 pg as cut off for iron deficiency within the first 24 h of life (14), others 30 pg (15). In the first months of life, some authors set the cut off at 27 pg, for both term and preterm infants (16, 17).

We recently conducted a survey on iron prophylaxis in Neonatal Units and NICUs of three Italian regions, underlining an important variability between the Centers in prescription of iron prophylaxis (dose, duration, type of iron). A few NICUs currently use CHr to modulate initial dose of iron supplementation in preterm babies and less than a quarter of late preterm babies received iron prophylaxis, affirming the need for practical guidelines for Neonatologists (18).

## Conclusions

Reticulocyte parameters are obtained on a very small sample of blood as part of the complete blood count and do not require additional phlebotomy.

ARC can distinguish anemia resulting from rapid destruction or loss of red blood cells from disorders causing bone marrow depression.

ARC and CHr are useful in the diagnosis of iron deficiency and iron deficiency anemia, in both children and newborn, and they are reliable and early predictor of response to oral iron therapy.

Reticulocyte parameters provide useful information to Pediatricians and Neonatologists for the diagnosis and the management of hematologic diseases. Don't forget to take advantage of them!

## Author Contributions

EP and FR gave substantial contribution to conception, drafted the article, reviewed and revised the manuscript. UR critically reviewed and revised the manuscript. All authors approve the final version of the manuscript as submitted and agree to be accountable for all aspects of the work.

## Conflict of Interest

The authors declare that the research was conducted in the absence of any commercial or financial relationships that could be construed as a potential conflict of interest.

## Abbreviations

ARC: Absolute reticulocyte count
CHr: Reticulocyte Hemoglobin Content
Hb: Hemoglobin
MCV: Mean Cellular Volume
NICU: Neonatal Intensive Care Unit.
